# How to have an acute gastroenteritis and an Anxiety Disorder at the same time: Cannabinoid hyperemesis syndrome (CHS) Case Series

**DOI:** 10.1192/j.eurpsy.2024.1177

**Published:** 2024-08-27

**Authors:** P. A. Hernández Liebo, J. Romay González, C. Sevilla Díez, O. S. Anabitarte Bautista, L. Cayón de la Hoz, G. E. Cortez Astudillo, M. Polo Gay, R. Obeso Menéndez, M. Hoyuelos Cob, M. Gómez Revuelta

**Affiliations:** ^1^Psychiatry, Hospital Universitario Marqués de Valdecilla; ^2^Psychiatry, Hospital de Sierrallana, Santander, Spain

## Abstract

**Introduction:**

Cannabinoid hyperemesis syndrome (CHS) is an underrecognized condition characterized by acute episodes of intractable nausea and vomiting, colic abdominal pain and restlessness related to chronic cannabis use. Antiemetics commonly fail to alleviate the severe nausea and vomiting. A very particular finding is the symptomatic relief with hot water. Antipsychotics (such as haloperidol), benzodiazepines and/or capsaicin cream appear to be the most efficacious in the treatment of this unique disorder. Precisely, it has been studied that transient relief of symptoms with topic capsaicin or hot water share the same pathophysiology. Nevertheless, abstinence from cannabis remains the most effective way of mitigating morbidity associated with CHS.

**Objectives:**

The objective is to study this phenomenom in our hospital and to alert of its existence in order to avoid a suspected misdiagnosis and overdiagnosis.

**Methods:**

We report a case series of seven patients who attended the Emergency Room (ER) of a third level hospital located in Cantabria (Spain) where a psychiatric evaluation was demanded.

**Results:**

The reasons for consultation were agitation and/or compulsive vomit provocation and showers. They were all women, with a median age of 29 years (range 21 to 38), who all smoked cannabis and in probable high doses (seven to up to twenty joints per day, information was missing in three of the patients) and probable long duration of consumption (more than nine years up to twenty-three, information was missing in three of the patients).

One of the most striking findings is the time to diagnosis, being the median of years of more than eight (range from two to twenty-one). In all of the cases there is a hyperfrequentation to the ER for this reason (not counting other emergency centres we have in Cantabria which we don´t have access to), being the average of almost twenty-two times (thirteen up to thirty times), not diagnosing it until last visits. Another interesting fact is that Psychiatric evaluation is done approximately in a third of the visits, being the department that makes all of the diagnosis except in one case. In all of the cases there are a lot of diagnostic orientation doubts from different medical departments, being the two most common psychiatric misdiagnosis: *Other Specified Anxiety Disorder* and *Other Specified Feeding or Eating Disorder.* Two of the patients were hospitalized in an acute psychiatric unit for this reason, one of them nine times and the other patient, twice.

**Conclusions:**

CHS has a very particular presentation which makes its recognition very simple. From our experience, it is an unknown entity for most of the doctors, something that needs to change in order to make a correct therapeutic management. Larger studies need to be done to make this findings more solid and for further information.

**Disclosure of Interest:**

None Declared

